# Supplementary material for: DNA end resection requires constitutive sumoylation of CtIP by CBX4
Source: Nat Commun. 2017 Jul 24;8:113. doi: 10.1038/s41467-017-00183-6 (PMC5524638; doi:10.1038/s41467-017-00183-6)
Supplement: Supplementary file 1 — Supplementary Information [file 41467_2017_183_MOESM1_ESM.pdf]

File name: Supplementary Information
Description: Supplementary figures, supplementary tables and supplementary references.

File name: Peer review file
Description:

## Supplementary Table 1: siRNAs

| siRNA | Sense sequence (5′–3′) | Reference | Supplier |
|---|---|---|---|
| **Non-target (NT) pool** | UGGUUUACAUGUCGACUAA, UGGUUUACAUCUUGUGUGA, UGGUUUACAUGUUUUCUGA, UGGUUUACAUGUUUUCCUA | D-001810-10 | Dharmacon |
| **CtIP** | GCUAAAACAGGAACGAAUC | [1] | SIGMA |
| **PIAS1** | GGAUCAUUCUAGAGCUUUA | [2] | SIGMA |
| **PIAS3** | CCCUGAUGUCACCAUGAAA | [2] | SIGMA |
| **PIAS4** | GGAGUAAGAGUGGACUGAA | [2] | SIGMA |
| **CBX4** | GUACUACUACCAGCUCAACUU | [3] | SIGMA |
| **hMMS21** | CAGAAGGAGUGGAUGAAGAUAUAAU | [4] | SIGMA |
| **RanBP2** | GAAGGAAUGUUCAUCAGGA | J-004746-12 | Dharmacon |
| **FUS** | GGACAGCAGCAAAGCUAUA | J-009497-09 | Dharmacon |
| **TRAF7** | CGGUGAAGCUGUGCUGUCA | J-007086-05 | Dharmacon |
| **UHRF2** | GUACUAUUGUCCCUUCUAA | [5] | SIGMA |
| **TOPORS** | CAAGGAGCCUGUCUAGUAA | [6] | SIGMA |

## Supplementary Table 2: Primers

| Primer name | Sequence (5′–3′) |
|---|---|
| **PIAS1 qPCR Fw** | CAGCTCTTCACCCAGTCCAT |
| **PIAS1 qPCR Rv** | CGCTGACTGTTGTCTGATGC |
| **PIAS3 qPCR Fw** | TGTCACCATGAAACCATTGC |
| **PIAS3 qPCR Rv** | AGGTAAAGTGCGCTTCCTCA |
| **hMMS21 qPCR Fw** | AAGCTGACGGAACAGAAGGAG |
| **hMMS21 qPCR Rv** | ATGGCGTCCTCTTCATAGGTG |
| **FUS qPCR Fw** | CTATGGAACTCAGTCAACTCCCC |
| **FUS qPCR Rv** | CTGCCCGTAAGACGATTGG |
| **TRAF7 qPCR Fw** | GTGTGGTGTCTCTGCGTCTAC |
| **TRAF7 qPCR Rv** | ACACAATGATGGTGCAGTCTG |
| **UHRF2 qPCR Fw** | GCCTTGGTGCTTGGTTTGAAG |
| **UHRF2 qPCR Rv** | AGACGTAGAGGGTACACTGTC |
| **TOPORS qPCR Fw** | CGACACCGACCTAGCTTTCT |
| **TOPORS qPCR Rv** | CCTTAGCAGCTGATGCCATT |

**Supplementary Table 3: Antibodies.** WB: western blot; PLA: proximity ligation assay; IF: immunofluorescence; IF-FISH: immuno-FISH

| Antibody | Species | Reference / Suppliers | Application |
|---|---|---|---|
| **CtIP** | Mouse | gift from R. Baer | WB (1:500)<br>PLA (1:250)<br>IF-FISH (1:50) |
| **GFP** | Rabbit | Santa-Cruz; sc-8334 | WB (1:1000) |
| **αTubulin** | Mouse | Sigma; T9026 | WB (1:50000) / IF (1:1000) |
| **PIAS4** | Rabbit | Abcam; ab58416 | WB (1:1000) |
| **CBX4** | Rabbit | Sigma; HPA008228 | WB (1:500) / PLA (1:250) |
| **RanBP2** | Mouse | Santa-Cruz; sc-74518 | WB (1:1000) |
| **γH2AX** | Mouse | Millipore; 05-636 | IF-FISH (1:250) |
| **γH2AX** | Rabbit | Cell Signaling; 2577L | IF (1:500) |
| **RPA32** | Mouse | Abcam; ab2175 | IF (1:500) |

| | | | |
|---|---|---|---|
| **RAD51** | Mouse | Abcam; ab213 | IF (1:1000) |
| **Cyclin A** | Rabbit | Santa-Cruz; sc-751 | IF-FISH (1:250) |
| **Histone H3** | Rabbit | Abcam; ab1791 | WB (1:20000) |
| **Biotin, fluorescein conjugated** | Goat | Vector Laboratories; SP-3040 | IF-FISH (1:250) |
| **BrdU** | Mouse | Amersham; RPN202 | SMART (1:500) |
| **Alexa Fluor 594 anti-mouse** | Goat | Invitrogen; A11032 | IF (1:1000), SMART (1:500) |
| **Alexa Fluor 488 anti-rabbit** | Goat | Invitrogen; A11034 | IF (1:1000) |
| **Alexa Fluor 568 anti-mouse** | Goat | Invitrogen; A11004 | IF (1:1000) |
| **Alexa Fluor 647 anti-rabbit** | Goat | Invitrogen; A21244 | IF (1:1000) |
| **Alexa Fluor 568 anti-rabbit** | Goat | Invitrogen; A11011 | IF-FISH (1:250) |
| **Alexa Fluor 647 anti-mouse** | Goat | Invitrogen; A21235 | IF-FISH (1:250) |
| **Alexa Fluor 568 anti-mouse** | Donkey | Invitrogen; A10037 | IF-FISH (1:250) |
| **Alexa Fluor 647 anti-goat** | Donkey | Invitrogen; A21447 | IF-FISH (1:250) |
| **IRDye 680RD anti-mouse IgG (H+L)** | Goat | LI-COR; 926-68070 | WB (1:5000 - 1:15000) |
| **IRDye 800CW anti-rabbit IgG (H+L)** | Goat | LI-COR; 926-32211 | WB (1:5000 - 1:15000) |

## Supplementary references


1. López-Saavedra, A. *et al.* A genome-wide screening uncovers the role of CCAR2 as an antagonist of DNA end resection. *Nat Commun* **7,** 12364 (2016).
2. Galanty, Y. *et al.* Mammalian SUMO E3-ligases PIAS1 and PIAS4 promote responses to DNA double-strand breaks. *Nature* **462,** 935–939 (2009).
3. Sakamoto, Y. *et al.* Overlapping roles of the methylated DNA-binding protein MBD1 and polycomb group proteins in transcriptional repression of HOXA genes and heterochromatin foci formation. *J. Biol. Chem.* **282,** 16391–16400 (2007).
4. Ni, H.-J. *et al.* Depletion of SUMO ligase hMMS21 impairs G1 to S transition in MCF-7 breast cancer cells. *Biochim. Biophys. Acta* **1820,** 1893–1900 (2012).
5. Oh, Y. & Chung, K. C. UHRF2, a ubiquitin E3 ligase, acts as a small ubiquitin-like modifier E3 ligase for zinc finger protein 131. *J. Biol. Chem.* **288,** 9102–9111 (2013).
6. Gibbs-Seymour, I. *et al.* Ubiquitin-SUMO Circuitry Controls Activated Fanconi Anemia ID Complex Dosage in Response to DNA Damage. *Mol. Cell* (2014). doi:10.1016/j.molcel.2014.12.001

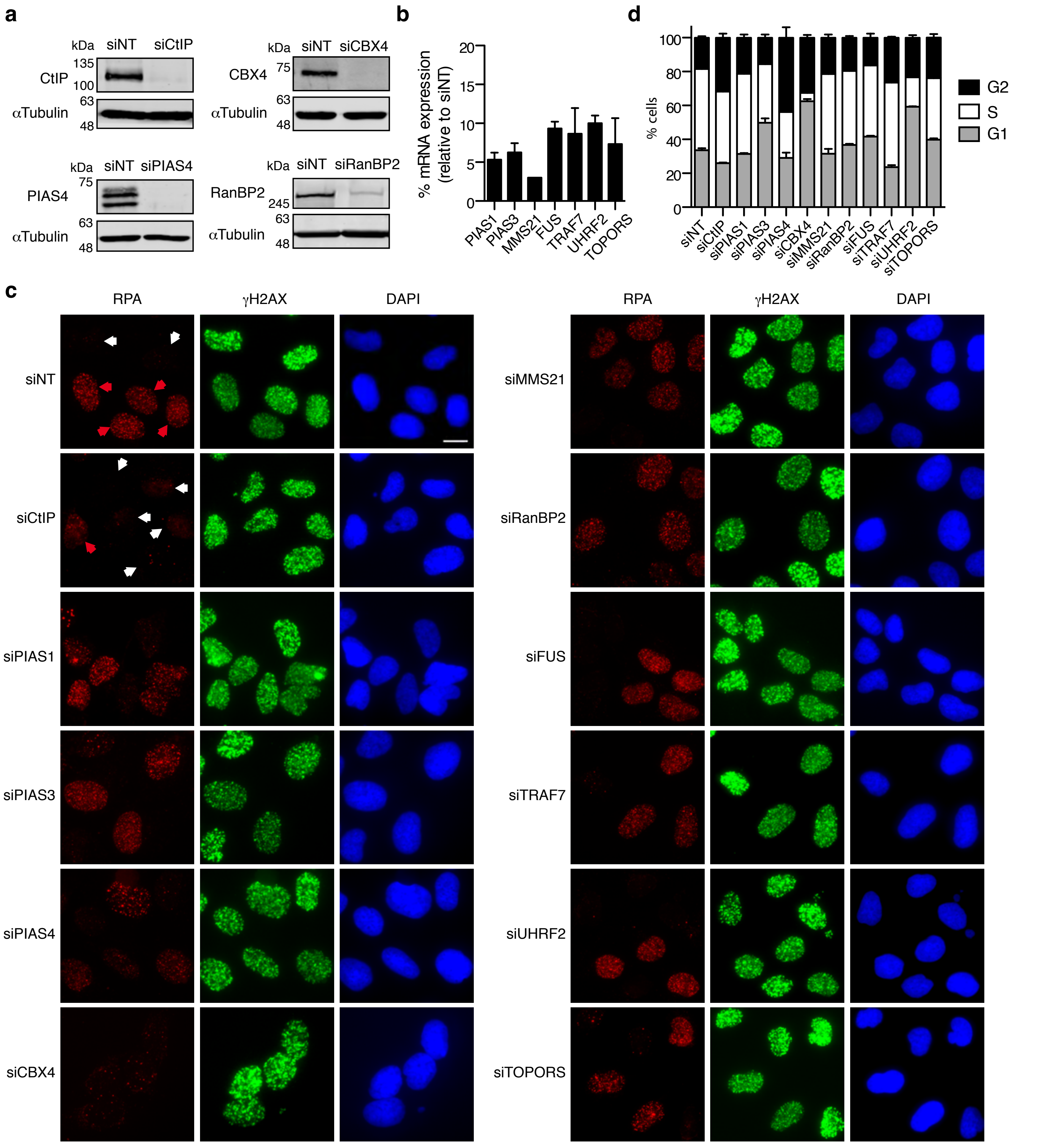


**Supplementary Figure 1. Effect of siRNA-mediated depletion of E3 SUMO ligases in cell cycle and RPA foci formation.**
(a) siRNA-mediated depletion of endogenous CtIP, CBX4, PIAS4 and RanBP2 shown by immunoblot. Protein extracts were obtained from U2OS cells 48 h after siRNA transfection. A representative experiment is shown. The white scale bar represents 15 µM. (b) siRNA-mediated depletion of endogenous PIAS1, PIAS3, hMMS21, FUS, TRAF7, UHRF2 and TOPORS mRNA showed by RT-qPCR. RNA was extracted from U2OS 48 h after siRNA transfection. Values represent the average and SEM of three independent experiments as the percentage of mRNA of each gene compared to siNT cells. (c) Representative fluorescence microscopy images of RPA foci (red) and γH2AX (green) in U2OS cells 48 h after transfection with the indicated siRNAs and 1 h after irradiation (10 Gy). Examples of cells negative and positive for RPA foci are marked with white and red arrows, respectively in the siNT and siCtIP panels. (d) Cell cycle distribution of U2OS cells 48 h after transfection with the indicated siRNA. The average and SEM of three independent experiments are plotted.

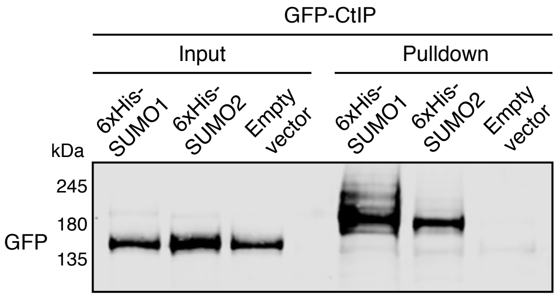


**Supplementary Figure 2. CtIP is modified by SUMO1 and SUMO2.**
Pulldown of 6 His-SUMO1 or -SUMO2 in protein extracts derived from GFP-CtIP–expressing HEK293T cells. Input represent 1% of the amount of protein loaded in the pulldown. Protein samples were resolved in SDS-PAGE and blotted with anti-GFP.

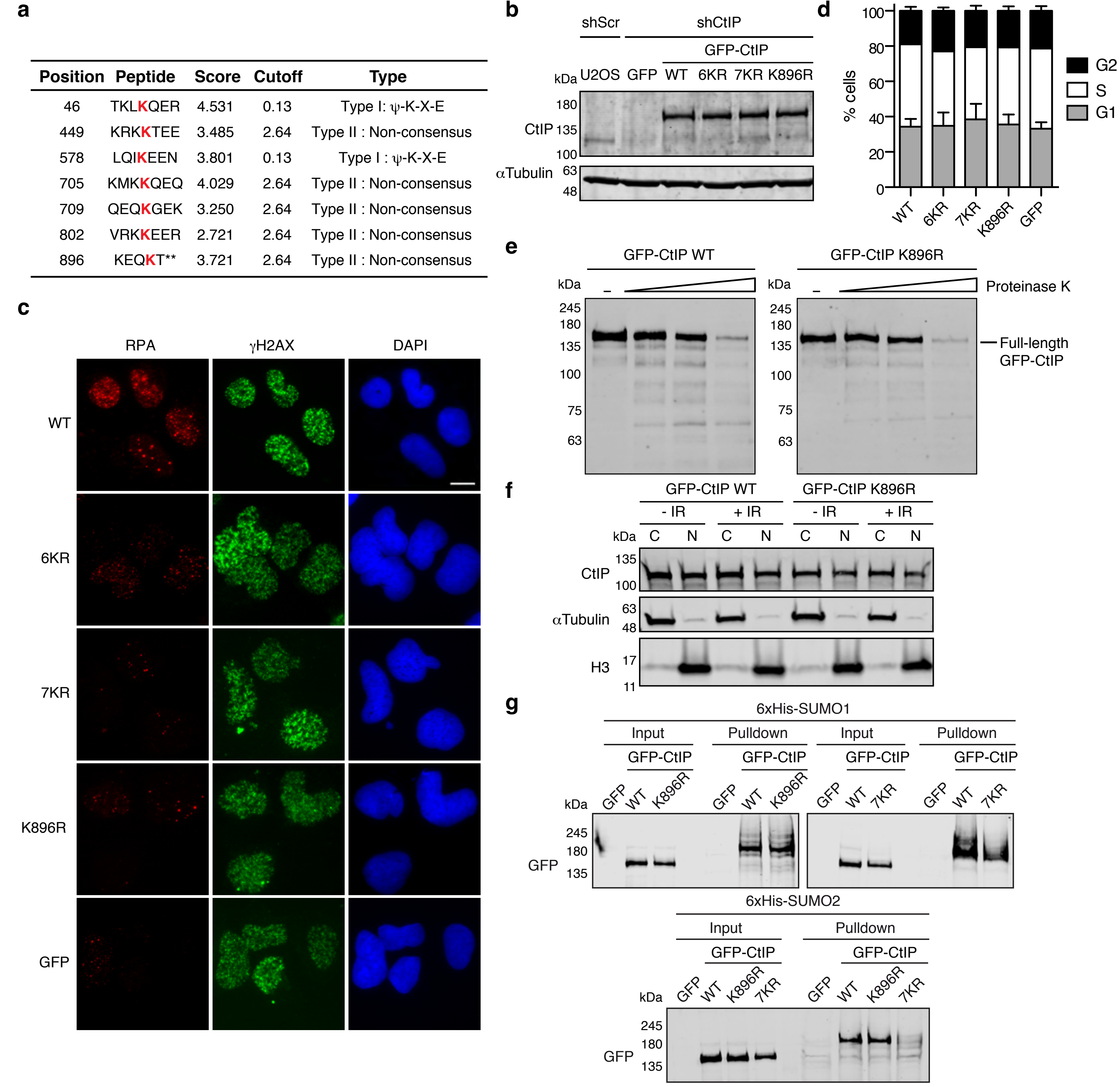

| Position | Peptide | Score | Cutoff | Type |
|---|---|---|---|---|
| 46 | TKLKQER | 4.531 | 0.13 | Type I: ψ-K-X-E |
| 449 | KRKKTEE | 3.485 | 2.64 | Type II : Non-consensus |
| 578 | LQIKEEN | 3.801 | 0.13 | Type I : ψ-K-X-E |
| 705 | KMKKQEQ | 4.029 | 2.64 | Type II : Non-consensus |
| 709 | QEQKGEK | 3.250 | 2.64 | Type II : Non-consensus |
| 802 | VRKKEER | 2.721 | 2.64 | Type II : Non-consensus |
| 896 | KEQKT** | 3.721 | 2.64 | Type II : Non-consensus |



**Supplementary Figure 3. CtIP sumoylation mutants.**
(a) Predicted sumoylation sites of CtIP provided by GPS-SUMOsp 2.0 software. (b) U2OS cells either expressing or not GFP-CtIP variants or GFP were transduced with shCtIP or shNT (non-target; used as a control) harbouring lentiviruses as indicated and selected with puromycin for 72 h prior to protein extraction. Protein samples were resolved by SDS-PAGE and blotted with the indicated antibodies. (c) Representative fluorescence microscopy images of RPA foci (red) and γH2AX (detected with a far-red secondary antibody but presented in green for better visualization) in U2OS cells expressing the indicated variants of GFP-CtIP or GFP, knocked-down for endogenous CtIP (as in b) and harvested 1 h after irradiation (10 Gy). The white scale bar represents 7.5 μM. (d) Cell cycle distribution of U2OS cells expressing the indicated version of GFP-CtIP or GFP and knocked down for the endogenous CtIP as detailed in b. The average and SEM of three independent experiments are plotted. (e) Proteinase K partial digestion of GFP-CtIP wild-type or the K896R mutant. GFP-CtIP was purified by GFP immunoprecipitation from protein extracts, and the resulting sample was divided into different aliquots and treated with increasing amounts of proteinase K to allow partial digestion, as indicated in the Methods section. Samples were then boiled and subjected to immunoblotting with an anti-CtIP antibody. (f) Cellular fractionation of U2OS cells expressing GFP-CtIP wild-type or the K896R mutant, irradiated (+IR) or not (–IR) at 10 Gy. C, cytoplasm; N, nucleus. See Methods for details. (g) Representative His pulldown experiment in cells expressing 6 His-SUMO1 (top) or 6 His-SUMO2 (bottom) in combination with either GFP or different GFP-CtIP variants as indicated. GFP-CtIP was detected using an anti-GFP antibody.

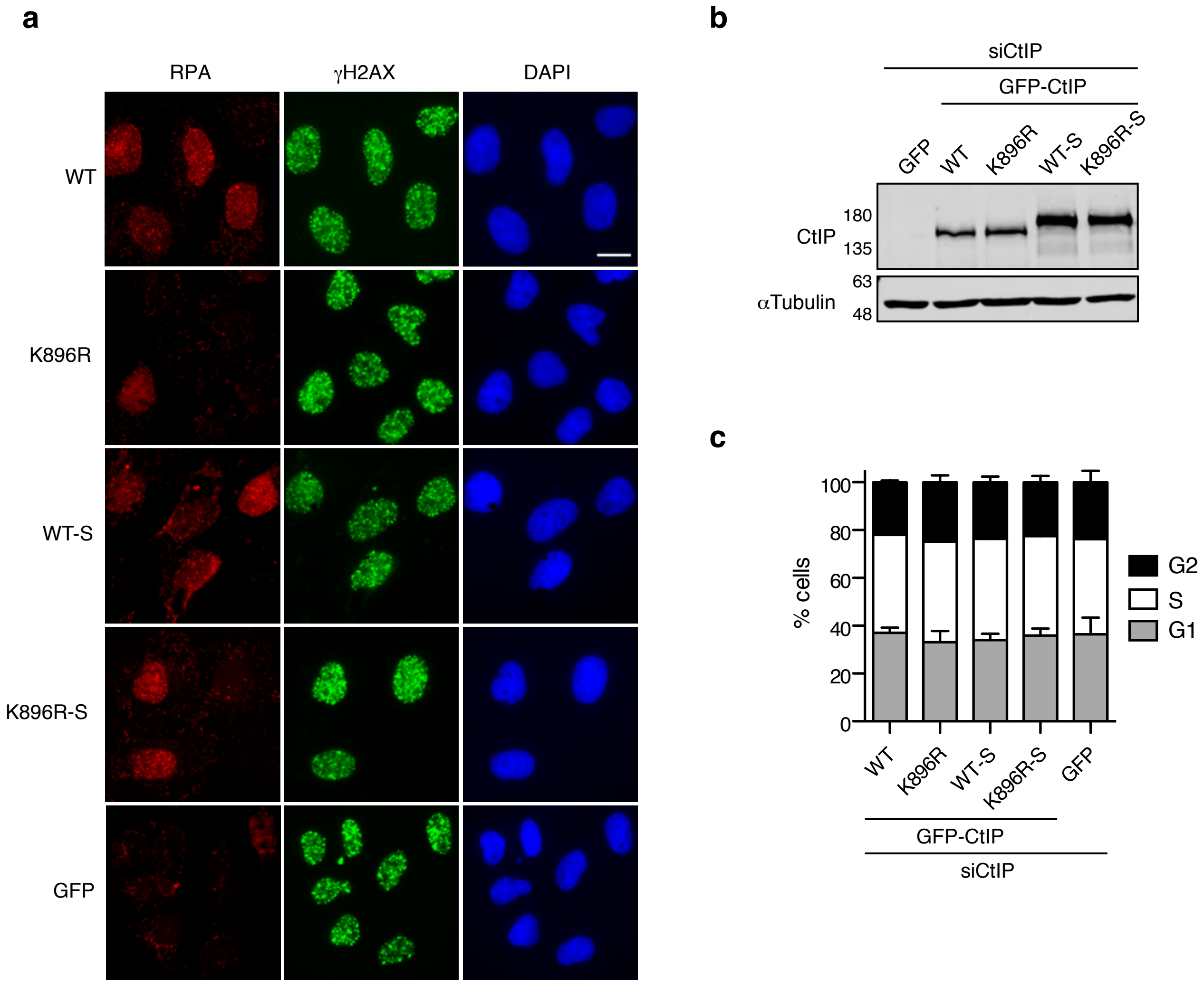


**Supplementary Figure 4. Expression of a constitutively sumoylated form of CtIP.**
(a) Representative fluorescence microscopy images of RPA foci (red) and γH2AX (detected with a far-red secondary antibody but presented in green for better visualization) in U2OS cells expressing the indicated variants of GFP-CtIP or GFP, knocked-down for endogenous CtIP by siRNA transfection and collected 1 h after irradiation (10 Gy). The white scale bar represents 7.5 μM. (b) Representative immunoblot analysis of GFP-CtIP expression corresponding to the cells shown in a using the indicated antibodies. (c) Cell cycle distribution of the cells shown in a and b. The average and SEM of three independent experiments are plotted.

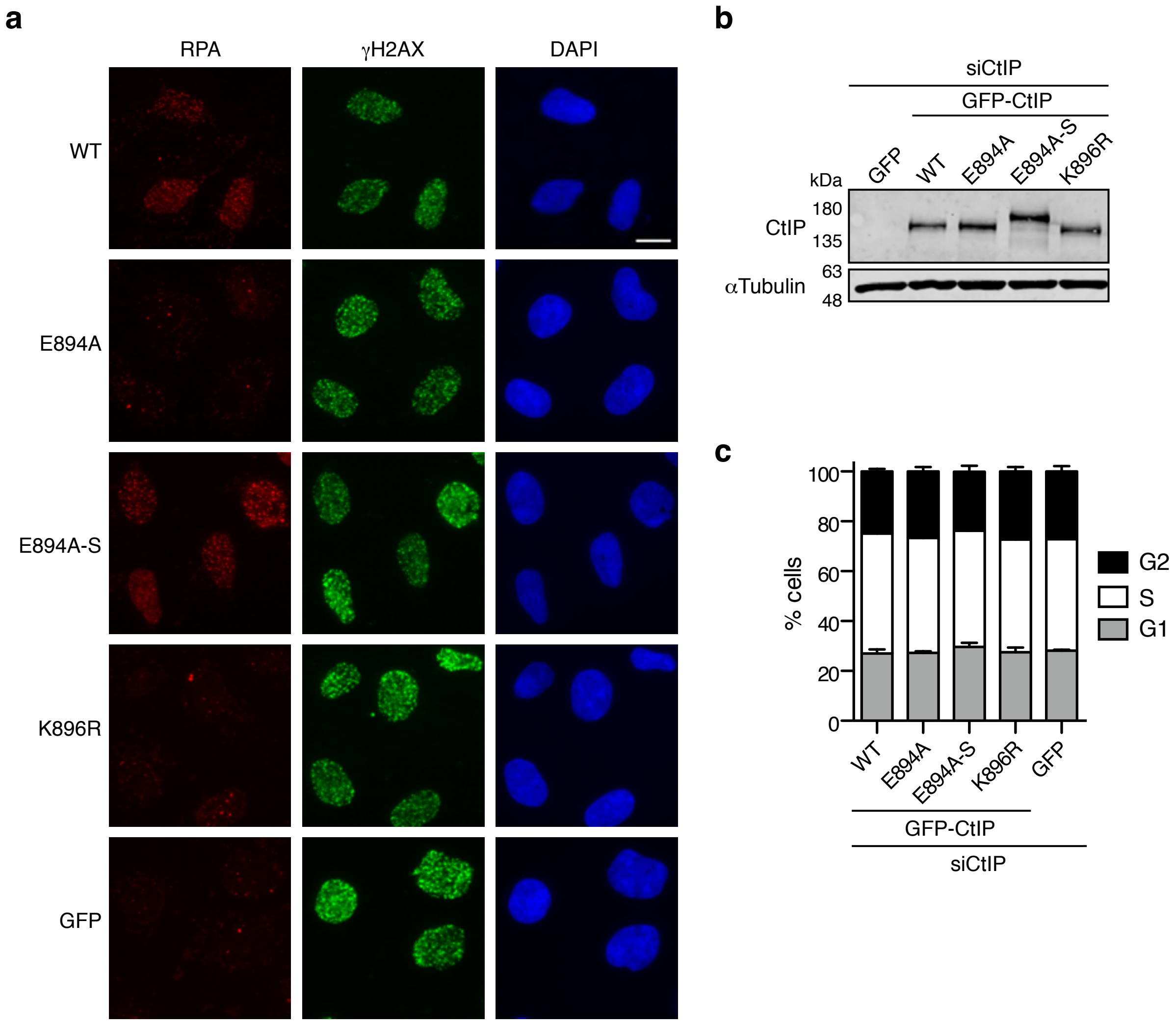


**Supplementary Figure 5. Impairment of sumoylation by an E894 mutation recapitulates K896 phenotypes**
(a) Representative fluorescence microscopy images of RPA foci (red) and γH2AX (detected with a far-red secondary antibody but presented in green for better visualization) in U2OS cells expressing the indicated variants of GFP-CtIP or GFP, knocked-down for endogenous CtIP by siRNA transfection and collected 1 h after irradiation (10 Gy). The white scale bar represents 7.5 μM. (b) Representative immunoblot analysis of GFP-CtIP expression corresponding to the cells shown in a using the indicated antibodies. (c) Cell cycle distribution of the cells shown in a and b. The average and SEM of three independent experiments are plotted.

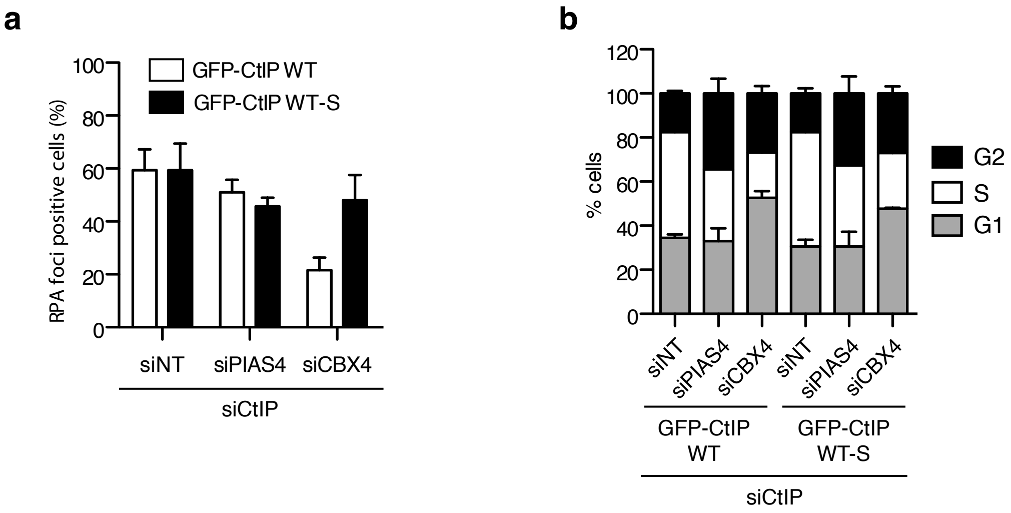


**Supplementary Figure 6. RPA foci raw data and cell cycle analysis in PIAS4 and CBX4-depleted cells in the presence of constitutively sumoylated CtIP.**

(a) Raw quantification of RPA foci positive cells in U2OS transfected with the indicated siRNA combinations and expressing wild-type GFP-CtIP or the constitutively sumoylated form of CtIP (GFP-CtIP-S) 1 h after exposure to 10 Gy of IR. Average and SEM of three independent experiments are shown. (b) Cell cycle distribution of cells treated as described in a.

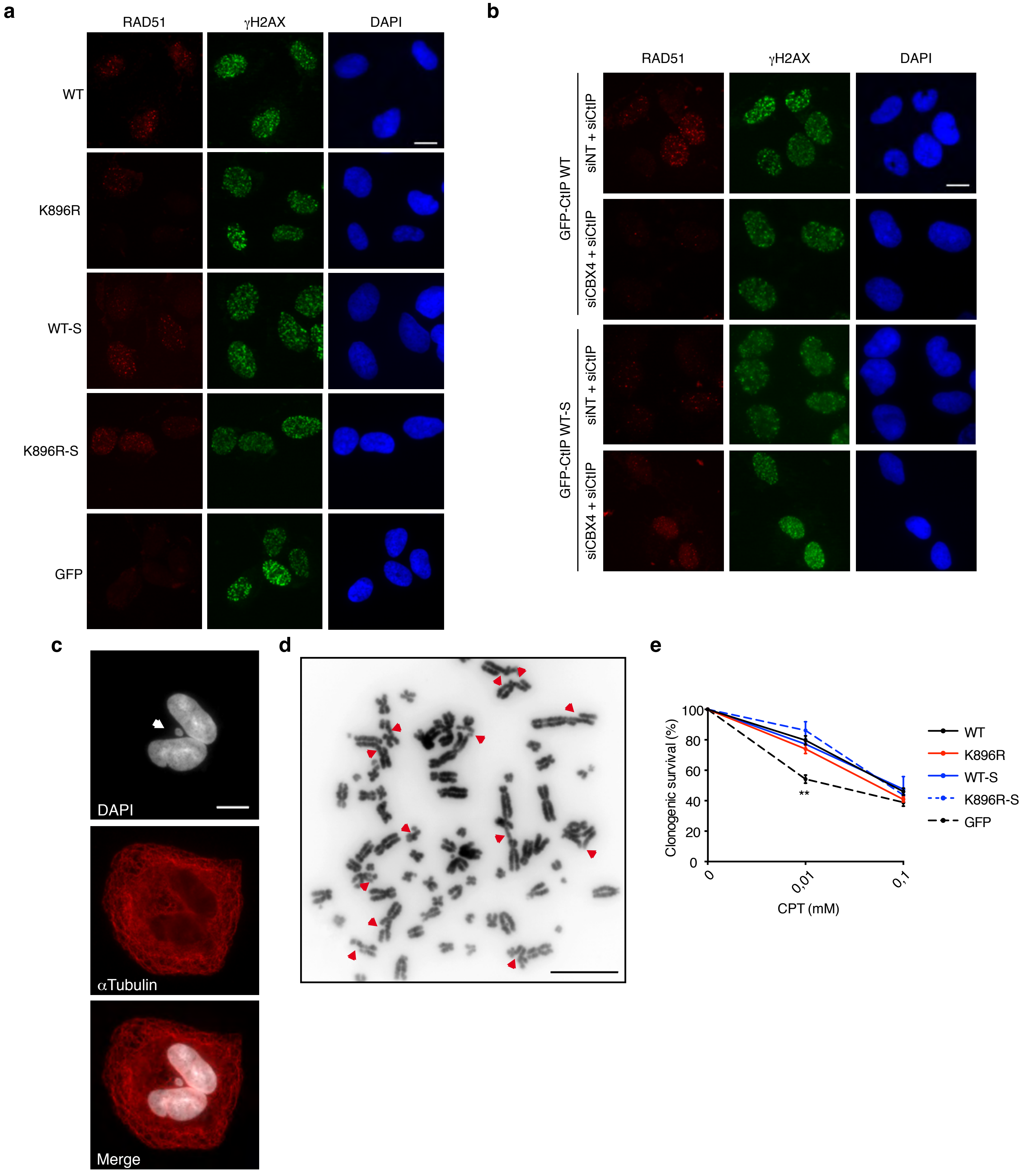


**Supplementary Figure 7. Impaired DSB repair in the absence of CtIP sumoylation**

(a) Representative fluorescence microscopy images of RAD51 foci (red) and γH2AX (detected with a far-red secondary antibody but presented in green for better visualization) in U2OS cells expressing the indicated variants of GFP-CtIP or GFP, knocked-down for endogenous CtIP by siRNA transfection and collected 3 h after irradiation (10 Gy). The white scale bar represents 7.5 μM. (b) Same as a, but in GFP-CtIP WT or WT-S transfected with the indicated combination of siRNAs. (c) Representative image of a micronucleus in a binucleated U2OS cell after IR. The white scale bar represents 7.5 μM. (d) Representative image of a mitotic spread showing different gross chromosomal aberrations (GCAs) (red arrows). The white scale bar represents 10 μM. (e) Clonogenic survival of GFP or GFP-CtIP expressing U2OS cells upon acute treatment with the indicated dosed of camptothecin. Other details as in figure 4e.

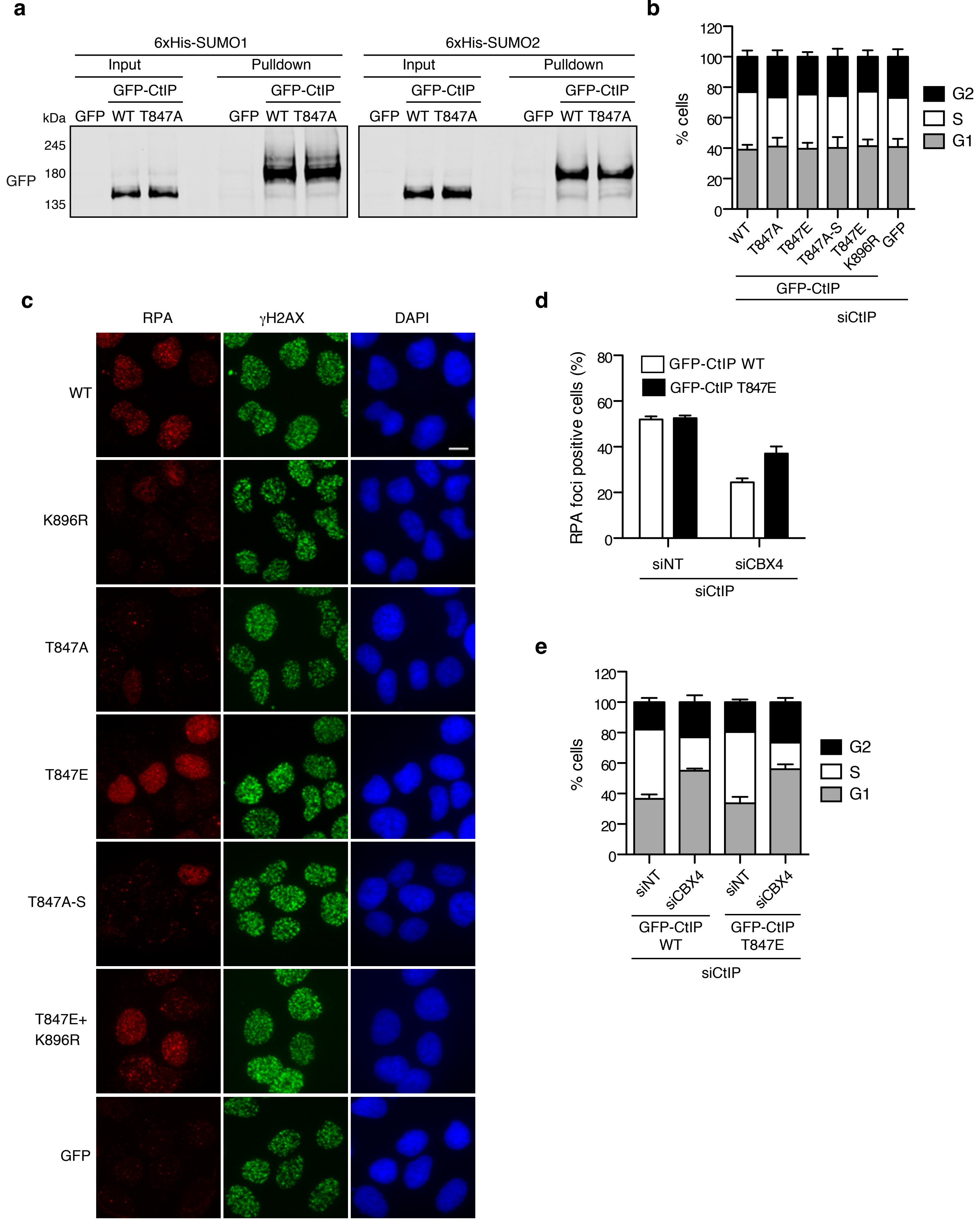


**Supplementary Figure 8. Connection between CDK-mediated T847 phosphorylation and sumoylation of CtIP.**
(a) Representative pulldown of 6 His-SUMO1 or -SUMO2 in protein extracts derived from HEK293T cells expressing GFP-CtIP wild-type or the non-phosphorylatable T847A mutant, or GFP as a control. Input represents 1% of the amount of protein used in the pulldown. Further details are as in Fig. 1e. (b) Cell cycle distribution of U2OS expressing the indicated form of GFP-CtIP or GFP, and knocked down for endogenous CtIP. The average and SEM of three independent experiments are plotted. (c) Representative fluorescence microscopy images of RPA foci (red) and γH2AX (detected with a far-red secondary antibody but presented in green for better visualization) 1 h after ionizing radiation (10 Gy) of cells treated as described in b. The white scale bar represents 7.5 µM. (d) Raw quantification of RPA foci–positive cells from U2OS cells expressing GFP-CtIP wild-type or the phosphomimetic T847E mutant and co-transfected with a combination of siCtIP and the indicated siCBX4 or siNT. DNA damage was induced with irradiation (10 Gy), and cells were collected 1 h later. Average and SEM of three independent experiments are shown. (e) Cell cycle distribution of the same cells as in d.

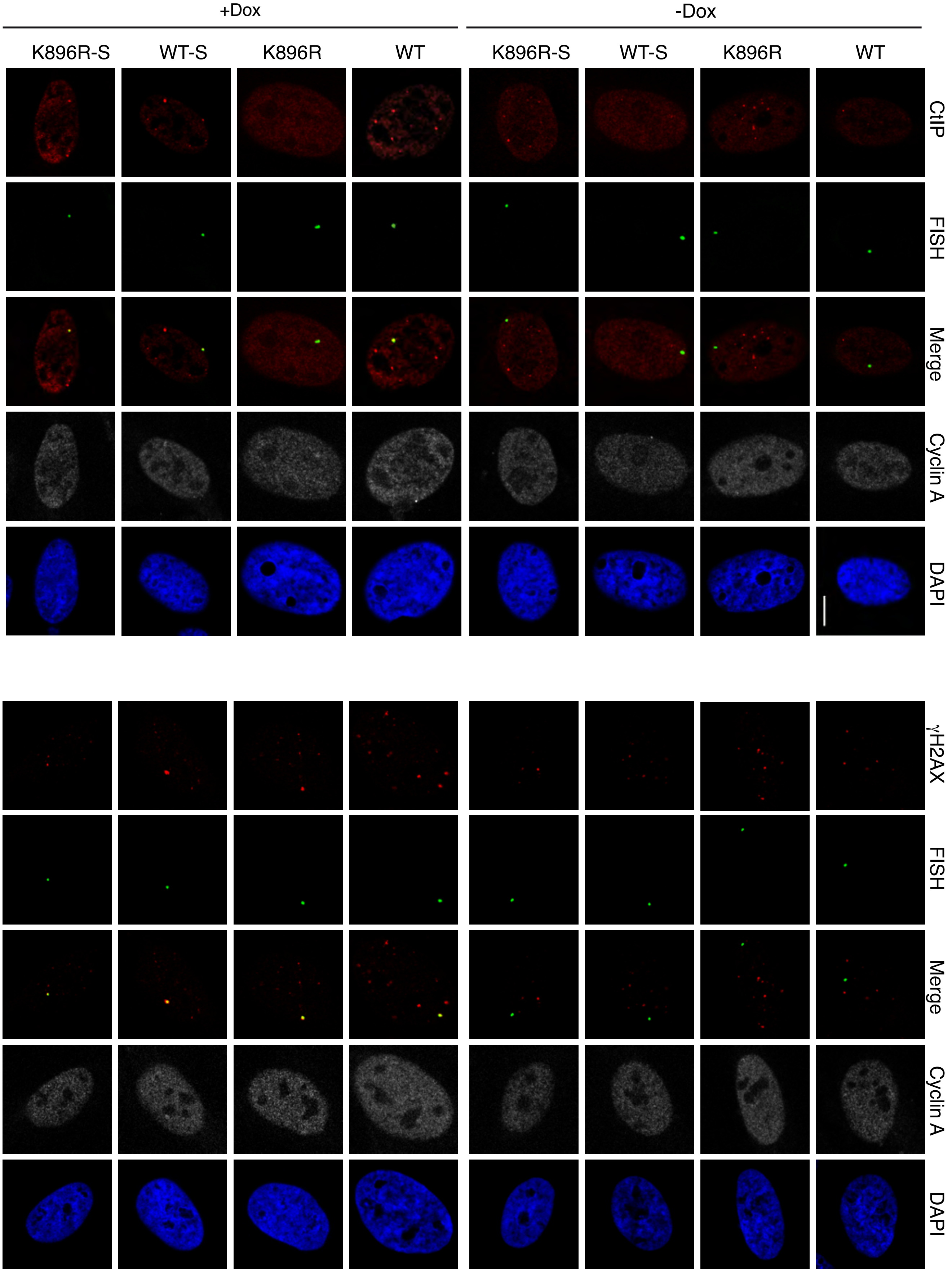


**Supplementary Figure 9. CtIP and γ-H2AX recruitment to I-SceI induced breaks.**
Representative images of CtIP (Top) or γ-H2AX (bottom) to a 256x LacO array in the absence (-DOX) or presence (+DOX) of an I-SceI induced double strand breaks in cells expressing different CtIP variants. The chromosomal location of the break was visualized with a FIS approach (green), whereas the proteins were observed using the appropriate antibodies (red). Cyclin A was visualized inthe far-red chanel to analyse cell cycle stage of thecells. See methods for details.
